# Supplementary material for: Desert Beetle-Inspired Hybrid Wettability Surfaces for Fog Collection Fabricated by 3D Printing and Atmospheric Pressure Plasma Treatment
Source: Biomimetics (Basel). 2025 Feb 26;10(3):143. doi: 10.3390/biomimetics10030143 (PMC11940326; doi:10.3390/biomimetics10030143)
Supplement: Supplementary file 1 [file biomimetics-10-00143-s001.zip › biomimetics-3475071-supplementary.pdf]

Supplementary Figures for

Desert Beetle-Inspired Hybrid Wettability Surfaces for Fog Collection Fabricated by 3D Printing and Atmospheric Pressure Plasma Treatment

Chia-Yi Lin [1], Ting-An Teng [1], Haw-Kai Chang [1,2] and Po-Yu Chen [1,]*

[1]*Department of Materials Science and Engineering, National Tsing Hua University, Hsinchu 300044, Taiwan*

[2]*Instrumentation Center, National Tsing Hua University, Hsinchu 300044, Taiwan*

[*]*Correspondence: poyuchen@mx.nthu.edu.tw*

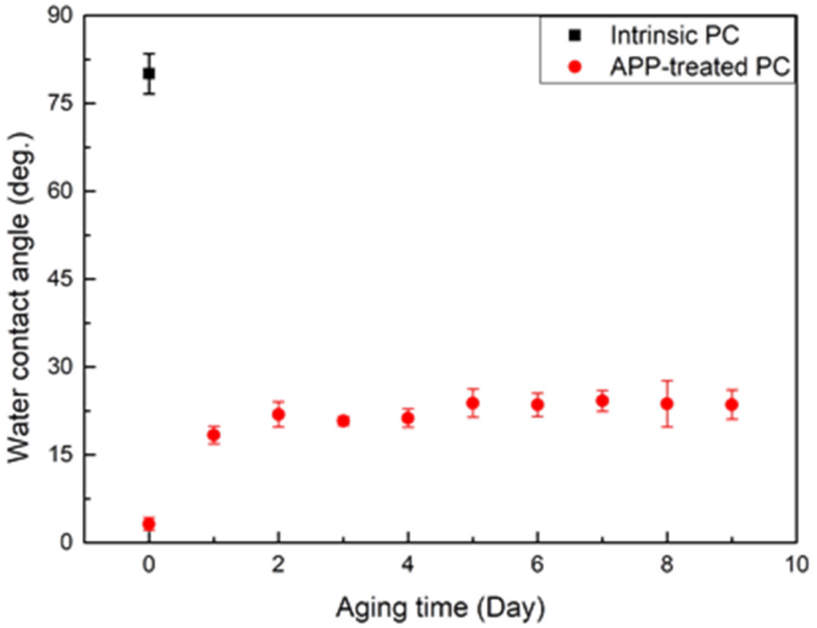

Figure S1. Water contact angles versus aging time of APP-treated PC for 9 days

The instant contact angle of the APP-treated PC was 3.2°. After one day, the contact angle increased to 18.4°. The contact angles were around 20° in the next few days.
